# The Probe for Renal Organic Cation Secretion (4-Dimethylaminostyryl)-N-Methylpyridinium (ASP+)) Shows Amplified Fluorescence by Binding to Albumin and Is Accumulated *In Vivo*

**DOI:** 10.1155/2022/7908357

**Published:** 2022-03-22

**Authors:** Jacob Schade Engbjerg, Vincenzo Costanzo, Donato Sardella, Luca Bordoni, Steen Jakobsen, Luciano D'Apolito, Jørgen Frøkiær, Francesco Trepiccione, Giovambattista Capasso, Sebastian Frische

**Affiliations:** ^1^Department of Biomedicine, University of Aarhus, Aarhus, Denmark; ^2^Department of Experimental, Diagnostic and Specialty Medicine, Alma Mater Studiorum-Università di Bologna, Bologna, Italy; ^3^Biogem Institute of Molecular Biology and Genetics, Via Camporeale, 83031 Ariano Irpino, Italy; ^4^Department of Nuclear Medicine and PET Center, Aarhus University Hospital, Aarhus, Denmark; ^5^Department of Translational Medical Science, University of Campania Luigi Vanvitelli, Naples, Italy

## Abstract

Accumulation of uremic toxins may lead to the life-threatening condition “uremic syndrome” in patients with advanced chronic kidney disease (CKD) requiring renal replacement therapy. Clinical evaluation of proximal tubular secretion of organic cations (OC), of which some are uremic toxins, is desired, but difficult. The biomedical knowledge on OC secretion and cellular transport partly relies on studies using the fluorescent tracer 4-dimethylaminostyryl)-*N*-methylpyridinium (ASP+), which has been used in many studies of renal excretion mechanisms of organic ions and which could be a candidate as a PET tracer. This study is aimed at expanding the knowledge of the tracer characteristics of ASP+ by recording the distribution and intensity of ASP+ signals *in vivo* both by fluorescence and by positron emission tomography (PET) imaging and at investigating if the fluorescence signal of ASP+ is influenced by the presence of albumin. Two-photon *in vivo* microscopy of male Münich Wistar Frömter rats showed that a bolus injection of ASP+ conferred a fluorescence signal to the blood plasma lasting for about 30 minutes. In the renal proximal tubule, the bolus resulted in a complex pattern of fluorescence including a rapid and strong transient signal at the brush border, a very low signal in the luminal fluid, and a slow transient intracellular signal. PET imaging using ^11^C-labelled ASP+ showed accumulation in the liver, heart, and kidney. Fluorescence emission spectra recorded *in vitro* of ASP+ alone and in the presence of albumin using both 1-photon excitation and two-photon excitation showed that albumin strongly enhance the emission from ASP+ and induce a shift of the emission maximum from 600 to 570 nm. *Conclusion*. The renal pattern of fluorescence observed from ASP+ *in vivo* is likely affected by the local concentration of albumin, and quantification of ASP+ fluorescent signals *in vivo* cannot be directly translated to ASP+ concentrations.

## 1. Introduction

Organic cations (OCs) are only slightly filtered during passage in the glomerulus due to their interaction with albumin or other plasma macromolecules. Renal excretion of OCs largely relies on secretion by the proximal tubule. At the basolateral membrane of proximal tubule cells, organic cation transporter 2 (OCT2) mediates diffusion-based uptake of a wide range of OCs down their electrochemical gradient, while at the apical membrane, OCs are secreted through the H^+^/cation exchange by multidrug and toxin extrusion proteins 1 and 2 (MATE1 and MATE2-K) [1, 2]. The transport system is far from fully understood but has for decades been recognized as remarkably efficient in clearing these molecules from the blood [3].

Of the naturally occurring OCs, some are listed as uremic toxins [4, 5], which through their accumulation may lead to the life-threatening condition known as uremic syndrome in patients with advanced chronic kidney disease (CKD) requiring renal replacement therapy (RRT) [6]. Although dialysis techniques constantly improve, the accumulation of these substances are still the main determinant of cardiovascular mortality in CKD patients [7]. It would thus be beneficial to developed means to assess and potentially stimulate the renal tubular secretory capacity in CKD patients [3]. In addition, the proximal tubular system for OC secretion is also responsible for the excretion of many drugs [2] and knowledge of the performance of renal tubular excretion is important to optimize the dosage and formulation of drugs [3]. There is therefore an unmet need for probes and other tools to assess the renal capacity for transport of organic cations in both *in vitro* and *in vivo* experimental systems and ideally in human patients, e.g., by PET imaging [8].

The organic cation 4-dimethylaminostyryl)-*N*-methylpyridinium (ASP+) was first defined as a probe for renal OC transport in experiments using the stopped flow technique in intact rat kidneys [9]. Since then, ASP+ has been proposed to be used for drug transport analysis [10] and widely used in various experimental settings [9–13]. Prior to the use in renal research, ASP+ was described as a fluorescent probe for living cells, which showed variable emission spectra depending on its interaction with different cellular structures [14] including mitochondria [15].

Our interest in ASP+ was stimulated by the single previous study of OC transport in the intact kidney using ASP+ and two-photon microscopy (2PM) of living rats [16]. In this previous study, ASP+ fluorescence appeared in peritubular capillaries 5 to 15 s after ASP+ injection. ASP+ fluorescence was then observed at the basolateral membrane of proximal and distal tubular cells followed by a peak of intracellular fluorescence with a long decreasing tail. ASP+ fluorescence decreased much slower in distal tubule cells than in proximal tubule cells. Moreover, the intracellular signal in proximal tubule cells was strongly affected by the presence of the OC cimetidine, indicating that the ASP+ signal reflected the OC transport capacity of the cells [17]. In addition, a blueshift in ASP+ fluorescence was observed at the basolateral membranes, and transientlyat the brush border in early segments of the proximal tubule.

We aimed to link experimental protocols based on ASP+ fluorescence to protocols based on ASP+ as a tracer for PET methodology, which would allow measuring of renal tubular secretory function at high spatial resolution in experimental animals using 2PM and PET imaging, which in contrast to 2PM holds translational potential to human subjects [8].

First, we reproduced previous findings using ASP+ and 2PM on the kinetics of the fluorescence signal for comparison with PET scanning of the tissue distribution and excretion kinetics using ^11^C-labelled ASP+.

Second, prompted by the observations of *in-vivo* 2PM experiments of:

- Almost absent ASP+ signal in the proximal tubular luminal fluid [16]
- A high-intensity fluorescence signal rapidly appearing in the brush border of proximal tubules
- A blue shift in ASP+ fluorescence [16]

we tested the hypothesis that ASP+ binding to albumin increases and blue-shifts the fluorescence signal.

## 2. Methods

### 2.1. Animals and Surgery

#### 2.1.1. Two-Photon Microscopy

Male rats of Munich Wistar Frömter (MWF) strain (age: 16 months, body weight (BW) 468–529 g, *N* = 4) were anesthetized with hypnorm (fentanyl 0.315 mg/mL, VetaPharma, UK) and dormicum (midazolam 5 mg/mL, Hameln, UK, MIDAZ210). A solution was made of 1 mL hypnorm, 1 mL dormicum, and 2 mL of sterile water. A subcutaneous injection of 0.3 mL/100 grams of BW was used as induction followed by 0.1 mL/100 grams of BW every 30–40 minutes.

A catheter (PE-50, Agntho's, Sweden) was placed in the external jugular vein, and a tracheostomy was made to facilitate spontaneous breathing. The left kidney was externalized and placed in a glass bottom dish, and the animal was covered with a heating blanket as described [16].

#### 2.1.2. PET Scanning

Male rats of the MWF strain (age: 14–16 months, BW: 478–507 g, *N* = 3) were anaesthetized in a chamber with 5% isoflurane in oxygen (O_2_) (0.4 L/min) and air (1.5 L/min). After induction of anesthesia, the head of the animal was positioned in a Plexiglas head holder and the anesthesia was maintained with a cone mask fitted to the head holder delivering isoflurane (1.8–2.0%) in O_2_ (0.4 L/min) and air (1.5 L/min). A transcutaneous catheter was placed in the tail vein for injection of ^11^C-ASP+. During the experiments, a heating pad was used to maintain body temperature and respiration frequency was monitored.

### 2.2. Fluorescent Plasma Dye/ASP+

#### 2.2.1. In Vivo Study

For 2PM, a stock solution of trans-4-[4-(dimethylamino)styryl]-1-methylpyridinium iodide (ASP+ (Sigma-Aldrich #336408-1G) was made from 1.2 *μ*moles of ASP+ and 0.8 mL phosphate-buffered saline buffer at pH 7.4 resulting in a concentration of 1.5 mM as done elsewhere [16].

#### 2.2.2. In Vitro Study

For the *in vitro* study, a stock solution of ASP+ was made from 0.036624 g of ASP+ dissolved in 500 mL of Krebs-Ringer buffer (KRB) resulting in a concentration of 200 *μ*M to be diluted subsequently. The KRB was composed of 20 mM HEPES, 135 mM NaCl, 5 mM KCl, 0.4 mM K_2_HPO_4_, 1 mM MgSO4, and 5.5 mM glucose, pH 7.4. For the experiments, a final ASP+ concentration of 10 *μ*M was used, except if indicated differently.

### 2.3. Radiochemistry

Cyclotron-produced ^11^CO_2_ or ^11^CH_4_ was converted to ^11^CH_3_I and directed to the reaction vial containing the precursor (1–1.5 mg of ASP+ in DMSO). After heating the reaction mixture for 5 min at 100 degrees, the crude product was diluted with 1 mL water and purified by reverse phase HPLC on a Luna C18(2) (5 *μ*m, 10 × 250 mm) (Phenomenex) column with 25% acetonitrile 75% aqueous NaH_2_PO_4_ (70 mM) as the mobile phase (flow 10 mL/min; *λ* = 254 nm). The fraction from the chromatographic separation corresponding to ^11^C-ASP+ was collected (retention time 8–9 min), diluted with water (20 mL), and trapped on a C18 Sep-Pak. After washing with 10 mL water, the product was eluted off with 1 mL ethanol followed by 9 mL saline into the final sterile product vial.

The radiochemical purity of the synthesized ^11^C-ASP+ was determined by analytical HPLC using an Ultimate® 3000 System (Dionex) (*λ* = 280 nm) connected to a GABI Star radio detector (Nuclear Interface). The chromatographic column was a Luna 5 *μ* C18(2) 100A (150 × 4.6 mm) (Phenomenex) with 30% acetonitrile 70% aqueous NaH_2_PO_4_ (70 mM) as the mobile phase (isocratic, 2.5 mL/min). The chromatographic data were analyzed using Chromeleon software (Dionex) (version 6.80). For all productions, the radiochemical purity was above 95%.

### 2.4. Two-Photon *In Vivo* Microscopy

Two-photon in vivo microscopy was performed on an upright Ultima IV two-photon microscope (Bruker, MS, USA) with a 20x objective (XLUMPlanFL20XW) NA 1.0, (Olympus, Japan), PrairieView software, and a Ti:sapphire laser (Chameleon Ultra II, Coherent, USA) operating at 800 nm supplemented by a converter arm (InverterScope, LSM Tech, USA) to allow inverted imaging. Emitted light between 570 and 620 nm was recorded using a Hamamatsu model 7422P-40 GaAsP detector. The frame size was set to 256 × 256 pixels and dwell time pr pixel to 0.8 *μ*s. The pixel size was 1.15 × 1.15 *μ*m and frame period 0.22 s.

An overview of the kidney cortex was made utilizing tubular autofluorescence since no fluorescent dye had been injected. For the imaging of ASP+ kinetics, a time series of 3 min with 1 frame per 0.22 seconds was recorded. After the first approximately 10 frames of the time series, a bolus of 0.2 mL of ASP+ stock solution was injected in the jugular vein using an automatized infusion pump (LUCCA Technologies, Harwinton, USA). Data was exported for further analysis as TIFF files.

### 2.5. Image Processing Using ImageJ

The *t*-series was denoised using the MATLAB implementation of V-BM4D [17] with default settings (“np” profile, Wiener filtering, sharpening, deflickering, and automatic noise estimation). To increase the execution speed of the algorithm in MATLAB 2018b (The MathWorks Inc., Natick, Massachusetts, United States), the single-threaded implementation of V-BM4D was executed in parallel by breaking the *t*-series in chunks having two overlapping frames at the end or at the beginning of each chunk. The total number of chunks was equal to the workers in the MATLAB parallel pool. The overlapping frames were removed from the denoised chunks and the sequence was reassembled before saving.

In order to compensate for sample drifting in the field of view, the *t*-series was opened in FIJI [18] and registered using the descriptor-based registration (2d/3d + t) plugin [19] with parameters left on default values saved for brightness of detections (medium), approximate size of detections (10 px), type of detections (maxima only), and transformation model (translation).

**Figure 1:**
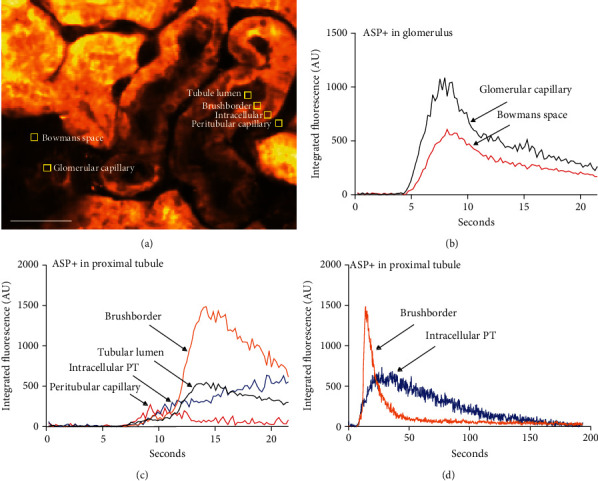
Quantitation of ASP+ signal appearance. (a) 6 ROIs (5 × 5 pixels) were placed in well-defined locations within the tissue. The ASP+ signal (after subtraction of background signal at *t* = 0) was recorded 5 times per second in each ROI for 21.5 s. Scale bar: 50 *μ*m. (b) Time course of ASP+ signal intensity in the ROIs located in renal corpuscle within the first 21 seconds. The signal increases in the capillary shortly earlier than in Bowman's space reflecting filtration of ASP+ from plasma to primary urine. The passage of the bolus is seen as a peak with a tail in both compartments. (c) Time course (initial 21 seconds) of the ASP+ signal intensity in the ROIs located in the proximal tubule. The signal almost simultaneously increases in the peritubular capillary and intracellularly in the proximal tubular cell. In the capillary, the signal appears as a small peak, whereas in the cell, the signal appears as a steady increase. A few seconds later, a strong signal appears in the tubular lumen and the brushborder. In these locations, the signal appears as a short peak with a tail. (d) Complete time course (app 3 min) of the ASP+ signal intensity in the ROIs located intracellularly and in the brushborder of the proximal tubules. The peak in the brushborder lasts much shorter than the peak of intracellular signal, which shows a long tail.

Fluorescence intensities were measured in FIJI by creating square regions of interest (ROIs) with a size of 5 × 5 px (see Figure 1(a)). The integrated fluorescence intensities in each ROI across the *t*-series were measured and stored in.csv files that were then imported in PRISM for generation of time courses shown in Figures 1(b)–1(d).

### 2.6. PET Scanning and Image Processing

Dynamic PET recordings using Mediso nanoScan PET/MR (Mediso Medical Imaging Systems, Budapest, Hungary) were initiated upon injection of a dose of ^11^C-ASP+. After the PET session, animals were decapitated.

The 60-minute dynamic PET scans were reconstructed with increasing duration from 10 seconds to 5 minutes (37 frames in all). PMOD version 3.5 (PMOD Technologies Ltd., Zurich, Switzerland) was used for imaging analysis. Multiple regions of interest were placed on coronal slices in the organ of interest creating a volume of interest (VOI); hence, time–activity curves from the heart wall, kidney cortex, and the liver were generated from the individual VOIs and data were expressed as standardized uptake values (SUV) by dividing the tissue concentration of ^11^C-ASP+ by the injected dose/weight of animal.

### 2.7. *In Vitro* Studies of ASP+ and the Effect of Bovine Serum Albumin

The effects of bovine serum albumin (BSA) on fluorescence properties of ASP+ were investigated. 96-well plates with ASP+ and BSA dissolved in KRB (300 *μ*L/well) were investigated with both 1-photon excitation (PerkinElmer 2300 Enspire, MS, USA) and two-photon excitation (Ti:sapphire pulsed laser attached to an investigator two-photon microscope (Bruker, MS, USA)). The Ti:sapphire laser power was 100 mW at the exit of the objective at all wavelengths. Emitted light was recorded in four wavelength intervals (435–485, 500–550, 570–620, and 640–680 nm) using the microscope detector module (frame size: 512 × 512 pixels, dwell time 2 *μ*s/pixel). The average signal from four frames was used. Background signals from wells containing milli-Q water were subtracted.

## 3. Results

### 3.1. ASP+ Enters the Proximal Tubule from Both Peritubular and Luminal Pathways

**Figure 2:**
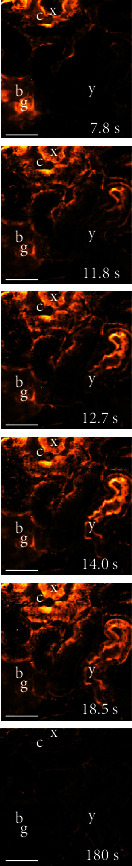
Time series showing the fluorescence signal in the kidney cortex of a bolus injection of ASP+. To visualize the passage of the injected bolus through the renal structures, the image recorded at *t* = 0 has been subtracted from the images recorded at each time point. 7.8 s: the bolus of ASP+ is visible in the glomerular capillaries (g), Bowman's space (b), and in some peritubular capillaries (c) as well as in the very basal part of cells in the proximal tubule (x) adjacent to capillaries with visible ASP+. Proximal tubules in areas of the cortex in which the bolus has not yet reached the capillaries or tubular lumen (y) do not show ASP+ signal at this time. Scalebars: 50 *μ*m .11.8 s: the proximal tubule labelled y begins to show basal ASP+ signal, and a strong ASP+ signal appears also in the brushborder of this proximal tubule. In the proximal tubule labelled x, the basal ASP+ signal now appears intracellular and diffuse. 12.7 s and 14.0 s: the strong ASP+ signal in the brushborder of tubule y spreads along the tubule and diffuse intracellular signal develops. 18.5 s: the strong ASP+ signal in the brush border of tubule y gradually diminishes but the diffuse intracellular signal remains. 180 s: the signal from the bolus of ASP+ is no longer evident.

After a bolus injection, the ASP+ signal appeared first in the glomerular capillaries and then Bowman's space (Figure 2, 7.8 s). ASP+ thereafter appeared in peritubular capillaries and consequently must be assumed to be available for absorption at the basolateral membrane of proximal tubular cells, in which a signal gradually appeared. In addition, a weak signal could be detected in the tubular lumen and a much stronger signal appeared at the brush border of the proximal tubule cells soon after the detection in the peritubular space (Figure 2, 11.8 s). Inspection of full-frame time series and videos indicated that the time course of the intracellular and luminal ASP+ signals differed and that signal in the brush border appeared transiently moving along the luminal surface (brushborder) of the cells (Figures 2, 11.8 s–14 s). Moreover, the signal in the tubule lumen appeared peculiarly weak relative to the signal in the brushborder. The signals in the lumen and the brushborder appeared short lived compared to the intracellular signal in the proximal tubule (Figure 2, 18.5 s). A signal could still be detected in the tissue some minutes after a single ASP+ bolus injection (Figure 2, 180 s). To see the effects of a single-bolus injection, a background subtraction for the fluorescence signal at *t* = 0 was performed for the images shown in Figure 2. It is evident that at *t* = 180 s, a single bolus of ASP+ leaves a small residual signal in the tissue. After several injections, the ASP+ signal accumulated and allowed the morphological identification of the tubular structures (Figure 1(a)). Recording of the integrated signal (*t* = 0 subtracted to see the effect of a single bolus) over time in subcellularly placed 5 × 5 pixel ROIs (Figure 1(a)) showed a clear peak of the ASP+ signal in Bowman's space immediately after ASP+ appeared in the glomerular capillaries, indicating filtration of ASP+ (Figure 1(b)). Similarly, the appearance of the ASP+ signal in the peritubular capillaries coincided with the appearance of an intracellular ASP+ signal in the proximal tubule (Figure 1(c)). The filtered bolus of ASP+ appeared slightly later in the lumen than in the adjacent peritubular capillaries. The luminal signal appeared simultaneously with a very strong peak-shaped signal in the brushborder, reaching a maximum 14 seconds after injection in the shown tubule cross-section (Figure 1(c)). The intracellular signal appeared first as a narrow line along the basal side of the proximal tubule but developed subsequently into a more diffuse intracellular signal. Thus, the intracellular signal increased slowly and peaked after 35 seconds and decreased over the following 150 seconds forming a much broader peak than the brushborder signal (Figure 1(d)).

### 3.2. ^11^C-Labelled ASP+ Accumulates in the Liver, Heart, and Kidney

**Figure 3:**
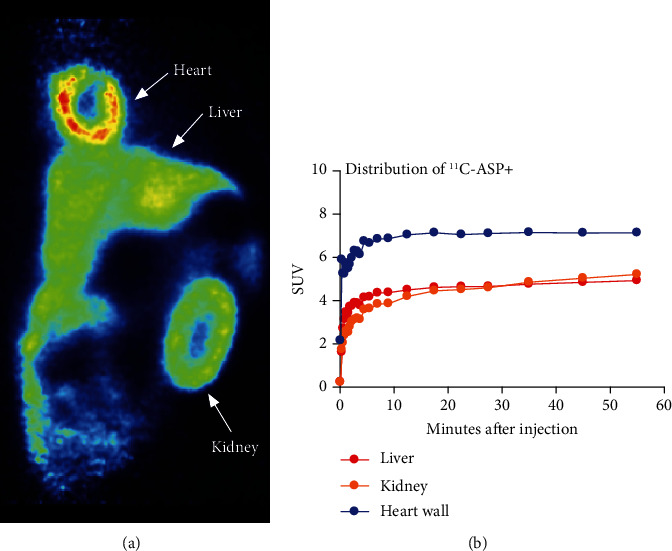
Whole body PET with ^11^C-ASP+.(a) Coronal image of PET summed over 60 minutes. (b) Time-activity curves for the heart wall, liver, and kidney cortex show accumulation of ^11^C-ASP+ in these organs. No decrease in ^11^C-ASP+ signal was seen during the scanning period.

A bolus injection of ^11^C-labelled ASP+ resulted in a strong accumulation of ^11^C radioactivity in the heart wall and a clear signal in the kidney cortex and the liver as evident from the summed PET image (Figure 3(a)). Time-activity curves for the heart wall, kidney cortex, and liver showed the accumulation of ASP+ to appear irreversible within the recording time of 60 minutes (Figure 3(b)).

### 3.3. Albumin Induces a Blue Shift of the Fluorescence Emission Peak of ASP+ from 605 nm to 570 and Increases the 2P Fluorescence Signal

**Figure 4:**
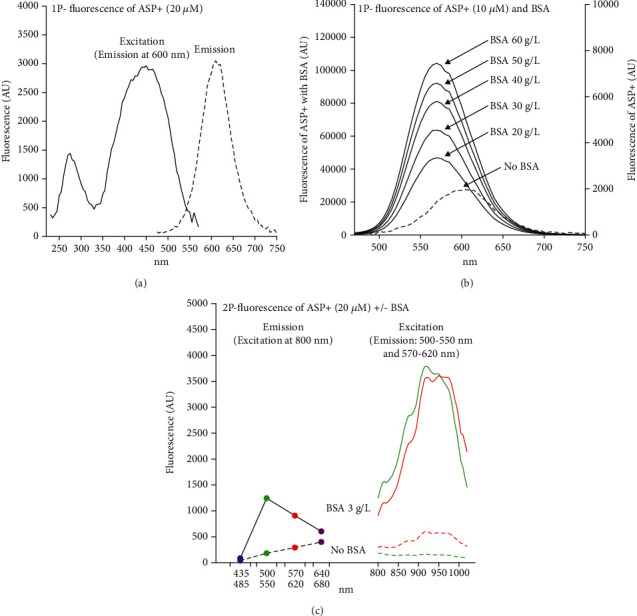
Fluorescence spectra of ASP+. (a) 1-photon fluorescence excitation spectrum and emission spectrum of ASP+. Maximum excitation was found at 450 nm (emission recorded at 600 nm), and maximum emission was seen at 605 nm. (b) The effect of BSA on the 1-photon fluorescence emission spectrum of ASP+. BSA induces a marked concentration-dependent increase in fluorescence (left *y*-axis) compared to ASP+ alone (right *y*-axis). In addition, BSA induces a blue shift of the emission peak from 605 to 570 nm. (c) Left: fluorescence emission from ASP+ in the absence (broken line) and presence of BSA (full line) in four wavelength intervals following excitation at 800 nm using a pulsed Ti:sapphire laser. Right: two-photon fluorescence excitation spectrum of ASP+ in the absence (broken line) and presence of BSA (full line). Maximum 2P excitation was obtained in the red channel (570–620 nm) at 920 nm and the emission from ASP+ alone was generally very low. BSA enhanced and induced a blueshift in the emitted signal.

The maximum 1-photon fluorescence emission of ASP+ is seen at 605 nm, and the maximum signal is reached using 450 nm light for excitation (Figure 4(a)). Addition of BSA to the ASP+ solution increases the fluorescence signal in a concentration-dependent manner and induces a shift of the emission peak to 570 nm (Figure 4(b)). Fluorescence emission from ASP+ after excitation by a pulsed laser operating at longer wavelengths typically used for *in vivo* imaging is rather modest, but maximum excitation was seen at 920 nm (Figure 4(c)). Addition of a small amount of BSA resulted in a multifold increase in 2P fluorescence signal at all excitation wavelengths, but the maximum excitation was still seen at 920 nm (Figure 4(c)). However, BSA induced a clear blue shift in the emission spectrum, since besides in the 570–620 nm interval, now, also a strong signal was evident in the 500–550 nm interval (Figure 4(c)).

## 4. Discussion

Investigating the mechanisms underlying the renal secretion of organic ions is fundamental to gather new knowledge on the renal secretion of some molecules acting as uremic toxins and some drugs. To this aim, the ASP+ fluorescent molecule has been previously used [9–13]. Besides the usage of ASP+ in kidney research, ASP+ has been widely used, e.g., to identify dopamine transporter-positive neurons *in vitro* [20] and serotonin transporter- (SERT-) expressing cells and to evaluate the activity and regulation of SERT *in vitro* [21]. In alveolar, bronchial, and intestinal epithelia, ASP+ has also been exploited to examine organic cation transporters [22].

In this study, we applied 2PM imaging to investigate renal proximal tubular handling of ASP+ in old MWF rats. Furthermore, we applied PET imaging of 11C-labelled ASP+, to link the experimental findings by 2PM to imaging modalities applicable to human subjects. Overall, we found a discrepancy between the results from the two methodologies, since PET imaging did not reveal any degree of excretion of an injected bolus of 11C-labelled ASP+, whereas the 2PM data showed a clear peak-shaped time course of ASP+ fluorescence in the proximal tubule. As PET imaging has much lower spatial resolution, this apparent discrepancy could be resolved by assuming ASP+ to be transiently present in the proximal tubule, but accumulating or at least excreted very slowly in other parts of the renal cortex, e.g., the distal tubules as previously reported [16].

### 4.1. ASP+ Signal Shows a Complex Pattern in the Proximal Tubule

Our 2PM data confirmed previous observations that ASP+ is taken up from the basolateral side of the proximal tubules [16]. However, our data question whether the basolateral uptake of ASP+ is the only path generating a signal in the lumen and brushborder of the proximal tubule. Indeed, we confirmed the appearance of a strong signal at the brush border of PT cells, which appeared to move as a bolus along the axis of the tubule. We therefore wondered whether a fraction of the injected bolus of ASP+ is filtered by the glomerulus and transiently adsorbed at the luminal side of the PT. Using a ROI-based quantitative analysis, we were able to detect an ASP+-dependent signal in the tubular lumen, albeit not easy to recognize on full-frame movies. However, if ASP+ is freely available in the plasma, we would expect it to be readily filtered due to the small size of the molecule. We were therefore still mystified that the ASP+ signal was so hard to detect in the lumen of the proximal tubule, especially since the signal at the brush border was so intense and appeared so rapidly.

The fluorescence of aminostyryl-pyridinium dyes like ASP+ is known to be sensitive to binding to lipid bilayers [23] but the emission spectrum of ASP+ did not show a blue shift compared to pure buffer when investigated in the presence of liver microsomes or artificial phospholipid microsomes [14], so lipid binding is unlikely to underlie the findings by *in vivo* 2PM using ASP+ [16]. Since we could not find a sufficient explanation in the literature for the complex time course and pattern of fluorescence signal in the lumen and brushborder of the proximal tubule following a bolus injection of ASP+, we considered other possible explanations.

As in the previous study employing 2PM [16], we used male rats of the Münich Wistar Frömter strain, but in contrast to the 250–300 g rats in the previous study, our rats were 16 months old and weighed >450 g. Since males of the MWF strain develop proteinuria with age [24–27], we speculated that this somehow could increase the intensity of the ASP+ signal in the lumen to a level where it could be detected. Based on the finding in our *in vivo* experiments of a detectable signal in the tubule lumen and a strong transient signal at the brush border (where filtered albumin is reabsorbed) and the apparent lack of excretion of ASP+ seen by PET imaging, we hypothesized that ASP+ can bind to macromolecules, in particular albumin, and that this binding enhances fluorescence emission. This would in turn imply that the recordable fluorescence signal by *in vivo* 2PM reflects a combination of ASP+ concentration and degree of binding to albumin, which could also contribute to resolve the discrepancy between our PET and 2PM data.

### 4.2. ASP+ Shows Strong 1P Fluorescence, Which Is Enhanced and Blue Shifted by the Presence of Albumin

Analysis of the properties of ASP+ by recording excitation and emission spectra after 1-photon excitation showed ASP+ to be a strong fluorophore, and in accordance with our hypothesis, the addition of albumin enhanced the signal. Moreover, a marked blue shift of the emission peak was evident upon interaction with albumin, which resembles the previous *in vivo* observations [16] of a blue-shifted ASP+ signal detected by increased emission below 550 nm in the brushborder of the proximal tubules.

### 4.3. ASP+ Shows Weak 2P Fluorescence, Which Is Drastically Enhanced and Blue Shifted by Albumin Binding

In contrast to our expectations of ASP+ to be suitable for 2P excitation, we found free ASP+ to be a rather weak fluorophore when excited for 2P fluorescence. However, as seen by 1P-excitation, the interaction with albumin markedly increased the emission signal of ASP+. Also, the blue shift induced by albumin binding was seen after 2P excitation (Figure 4(c)).

### 4.4. The Interaction between ASP+ and Albumin Determines the Pattern and Time Course of the Fluorescence Signal *In Vivo*

With the knowledge that ASP+ bound to albumin gives a much stronger fluorescence signal than ASP+ alone, we propose the following explanation of the pattern and time course of the fluorescence signal in the renal cortex following a bolus injection of ASP+:

1. In blood plasma, a strong signal is seen due to the presence of albumin, which binds ASP+
2. In the ultrafiltrate, the ASP+ signal is undetectable in young rats [16], but detectable in old MWF rats in the present study, since these rats loose albumin to the ultrafiltrate [24–27]
3. The filtered fraction of albumin is bound for reabsorption at the brush border, and ASP+ albumin complexes could be expected to appear in higher concentration at the brushborder than in the bulk tubular fluid, which explains the rapidly appearing strong apical signal in the brushborder. The intensity of the brushborder signal has previously been seen to fall with the distance from the glomerulus [16], which is also consistent with the signal to depend on albumin, since albumin is preferentially taken up in the initial part of the proximal tubule, and thus, less albumin is available further down the tubule
4. It is unclear if the filtered ASP+ is reabsorbed together with albumin or may dissociate from albumin before reabsorption. After reabsorption, albumin is transported to lysosomes for breakdown but no ASP+ signals reminding of lysosomal structures were seen, indicating ASP+ to not follow albumin. On the other hand, the fluorescence signal from reabsorbed albumin ASP+ complexes would be drastically reduced when albumin is broken down
5. At the basal side of the proximal tubule, the ASP+ signal appears concomitantly with the bolus in the adjacent peritubular capillaries. Subsequently, an intracellular ASP+ signal appears in the proximal tubule cells. This is in accordance with ASP+ being taken up for secretion as other organic cations
6. Since free ASP+ gives a relatively weak 2P fluorescence signal and since albumin is not taken up basolaterally, the longer-lasting intracellular ASP+-dependent signal may reflect that other proteins or macromolecules can bind ASP+ and, similarly to albumin, induce an enhanced 2P fluorescence of ASP+. The intracellular fluorescent signal diminishes over minutes, which may reflect a breakdown of such 2P-fluorescent complexes, which may allow secretion of free ASP+ as previously proposed and supported by the competitive inhibition of the signal reduction rate by cimetidine [16]. The residual signal, which leads to accumulation of the background signal, may result from long-lasting binding of ASP+ to intracellular proteins or lipid bilayers of membranes in cell organelles. However, ASP+ has also previously been shown to accumulate in mitochondria [15] and this mechanism may underlie the previously seen more persistent presence of the fluorescent ASP+ signal in the mitochondria-rich distal tubules

## 5. Conclusion

Due to its interaction with albumin, ASP+ is unlikely to be a useful probe for *in vivo* studies of epithelial transport and secretion processes of organic cations and care should also be taken when interpreting data from albumin-free *in vitro* systems, since the ASP+ signal cannot be excluded to be influenced by binding to other proteins. Further characterization of the interaction between ASP+ and albumin may more precisely specify the limitations for the use of ASP+ and may possibly allow ASP+ to be used as a highly fluorescent probe to visualize albumin both *in vitro* and possibly *in vivo*.

## Data Availability

Data are available from the corresponding author upon reasonable request.
